# The Dropsy of Popes (1555–1978): A Bad Prognostic Sign Foreboding of Death

**DOI:** 10.1007/s10943-022-01578-6

**Published:** 2022-05-20

**Authors:** Natale Gaspare De Santo, Carmela Bisaccia, Luca Salvatore De Santo

**Affiliations:** 1 Università Della Campania Luigi Vanvitelli, Naples, Italy; 2 Mazzini Institute Naples, Naples, Italy; 3 Division of Heart Surgery, University of Campania Luigi Vanvitelli, Naples, Italy

**Keywords:** Edema, Popes, Chronic kidney diseases, Salt consumption

## Abstract

The purpose of this study is to explore the historical background of edema as a prognostic sign in popes, a special category of medical subjects whose health status was closely monitored and chronicled because of their unique important status in the events of their times. Nine out of 51 popes, who reigned in the years 1555–1978, died edematous at a mean age of 75.5 years of age. The cause of edema was: heart failure for John Paul I, liver disease, obstructive nephropathy associated with anemia for Paul IV, who also suffered from deep vein thrombosis, and malnutrition for Innocent XIII. Chronic kidney disease due to renal stones of gouty origin caused edema in Clement VIII, Clement X, Clement XI, and Benedict XIV. Obstructive nephropathy due to renal stones of non-gouty origin caused edema in Clement XIII, whereas toxic nephropathy due to the use of mercurials caused edema in Clement XIV. Innocent XI, Benedict XIV, and Clement XIV were bled before death because of impending pulmonary edema. It is not surprising that chronic kidney disease was a significant cause of edema in popes with chronic kidney disease which is associated with impaired sodium excretion. The edema was likely aggravated by the excessive dietary salt intake of the period when the importance of sodium chloride restriction was still not discovered and effective diuretic agents were not available.

## Background

Edema from the Greek stem *Oidêma* is defined as an accumulation of fluid in the interstitial compartment of organs and tissues, often due to an imbalance of the circulatory forces controlling local interstitial fluid exchange (Renkin and Chrone, 1986). Edema may be localized as in inflammation, venous, or lymphatic insufficiency or generalized as in cardiac failure, cirrhosis of the liver, nephrotic syndrome, acute and chronic renal failure, toxemia of pregnancy, and idiopathic (De Santo et al., 1997).

The problem of edema formation and resolution is as old as the practice of medicine itself. It is recorded in the Ebers Papyrus (about 1522 BC) and the Berlin Papyrus (ca. 1300 BC). In the *Corpus Hyppocraticum (460–370 BC)*, edema (*oidêma),* dropsy (*udrôps)*, and anasarca (dropsy throughout the flesh = *ana sarx)* were considered “a bad sign …” that is difficult to treat and sometimes “fatal” (Touwaide & De Santo, 1999). It was only in 1896 that an integrating hypothesis attributing edema to altered capillary forces of fluid exchange was advanced (Starling, 1896). A unifying hypothesis of the role of sodium chloride in body fluid volume regulation in health and the edema of disease and its treatment was advanced by Robert Schrier (1936–2021) (Elhassan & Schrier, 2010; Schrier, 1988). Still, edema remained an ominous sign that was poorly controlled until the last century when organic mercurials were introduced (Vogl, 1915). They were followed from 1950 onward by the discovery of more effective oral diuretics such as acetazolamide, chlorothiazide, ethacrynic acid, and furosemide (Eknoyan, 1997; Pitts, 1959).

The aim of the present study is to explore the historical prognosis of edema in the elderly, an important and yet neglected clinical subject (Thaler et al., 2010), by examining the occurence of dropsy in popes as a representative of the elderly over the past four centuries. Since the Renaissance, in the period 1493–2005, there have been 51 popes who started their pontificate at a mean age of 63.9 years, reigned for an average of 10 years and died at a mean age of 73.6 years (Retieff & Cilliers, 2005). Nineteen of them were older than 80 years at death. Popes represent a special category of medical subjects whose health status has been closely monitored and chronicled because of their uniquely important position in the history of their times(De Santo et al., 2020, 2021a, 2021b). Papal dropsies, their etiology, and the prognostic value of edema were examined in the papal histories from Boniface VIII to John Paul II, a span of over seven centuries (1294–2005). Data will be presented on a group of 9 popes—reigning in the period 1555–1978—in whom overt fluid retention presented as a poor prognostic sign that was a harbinger of death in 8 of them.

## Methods

We have studied the narratives of 9 dropsical popes, Paul IV, Innocent IX, Clement VIII, Clement X, Innocent XI, Innocent XIII, Benedict XIV, Clement XIV, and John Paul I (Table 1). Their narratives were compiled from the account of the papal histories of De Novaes (1822–1825), Gualino (1934), von Pastor (1958–1964), Ceccarelli (2001), Retieff and Cillliers (2005), and Cosmacini (2018). The long-standing disease of John Paul I was studied in the biography of Roncalli (2012).

**Table 1:** Life-span of edematous Popes. Birth date, duration of pontificate and age at start and at the end of pontificate

Pope	Birth	Pontificate	Age at start	Age at death
Paul IV	1476	5/13, 1555–8/28, 1559	61	85
Innocent IX	1519	10/29, 1591―12/30, 1591	73	73
Clement VIII	1535	1/3, 1592―3/3, 1605	57	70
Clement X	1590	4/29, 1670―7/22, 1676	80	86
Innocent XI	1611	9/21, 1676―8/12, 1689	65	78
Innocent XIII	1655	8/17, 1721―3/7, 1724	66	69
Benedict XIV	1675	8/17, 1740—5/3, 1758	65	83
Clement XIV	1705	5/719, 1769―9/22,1774	64	69
John Paul I	1912	8/26, 1978―9/28, 1978	66	66

## Narratives of Dropsical Popes

(I) Paul IV, Pontificate: 23 May 1555–28 August 1559.

**Fig. 1:**
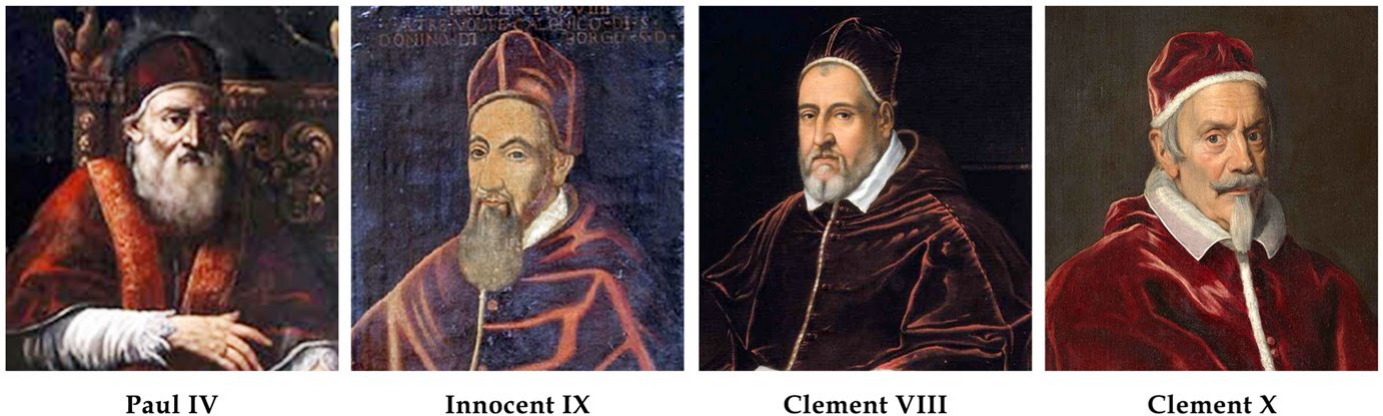
Popes reigning from 1555 to 1676

Born GianPietro into the Neapolitan branch of the Carafa family, in the town of Sant’Angelo della Scala Avellino on 28 June 1476. He was 79 years old at the beginning of his papacy. He is mostly remembered for having established the Jewish Ghetto in Rome (12 July 1555), for the *Index Librorum Prohibitorum* (1559) and for recruiting from Padua the anatomist Mateo Realdo Colombo (c.1516–1559) as *archiater pontificalis* and professor at the University Sapienza in Rome where he taught until 1559 (Fig. 1).

Paul IV was a stoutly built man who started his pontificate in good health. He loved lavish meals but was a sober eater, fasting regularly and abstaining from meat, but accompanied his meals with a glass of dark Neapolitan wine or some Malvasia.

The Pope was old, suffered from gravel, chronic kidney disease and probably anemia.

Also, according to Ceccarelli (2001) he had liver disease of unknown origin. He was likely hydropic due to impaired renal function due to stone disease and secondary to his liver disease. His legs were edematous and testicles swollen. He was always cold and even in summer had to heat his residential quarters. Old age and anemia could also explain his sense of cold. He was treated with purges and given rose syrups and other simples to reduce edema. He died in the afternoon of August 18 1539.

(II) Innocent IX, Pontificate: 29 October 1591–30 December 1591.

Born Giovanni Antonio Facchinetti in Bologna in July of 1519. He studied law and earned a doctorate. At the age of 25, he moved to Rome where he worked for Cardinal Farnese. He was nuncio to the Republic of Venice, and as bishop participated in the council of Trent. He became a Cardinal in 1583 (Fig. 1).

An ascetic and scholarly man of frail constitution, he was elected pope at the age of 72 years. Reports indicate that he was a frugal eater and a sober drinker who fasted regularly (De Novaes, 1822–1825). At breakfast he would have a bowl of barley soup and went on working throughout the day with a short break for a walk in the Vatican Gardens. His health was likely frail as he is described as a “skinny person, the shadow of a man”. He developed leg edema for the first time on 6 November 1591, 55 days before death (von Pastor, 1958–1964). Later he is said to have a cold without fever but was obliged to stay in bed because of his weak constitution. He probably died from an acute pneumonia that was superimposed on pulmonary edema (Reardon, 2004) on 30 December (De Novaes, 1822–1825; von Pastor 1958–1964).

(III) Clement VIII, Pontificate: 30 January 1592–3 March 1605

Born Ippolito Aldobrandini on 24 February 1535 at Fano in Tuscany and elected pope in 1592 (Fig. 1). He is remembered for the Catholic Reformation in many countries, updating the *Index Librourum Prohibitourm*, and specially for having condemned the philosopher Giordano Bruno to be burned at the stake on 17 February 1600.

He is known for building the Villa Aldobrandini in Rome, for his support of the arts and sciences and for his intellectualism. The botanist and philosopher Andrea Cesalpino (1524–1603) became his personal physician and was nominated *professore straordinario* at the University Sapienza in Rome. Cesalpino is remembered for his *Questionum Peripateticarum Libri V* and *Quaestionum Medicarum Libri II* where he stated that the heart was the center of blood flow, not the liver as professed by Galen, and introduced the term *circulatio sanguinis*.

Clement was tall, robust, and stately. For exercise, he enjoyed walking through the Vatican gardens. His first symptoms of gout were manifested in April 1595. Lorenzo Gualino (1934) reported it in a letter to the Senate of Venice. The Venetian historian Paulo Paruta (1540–1598) wrote that the illness was probably associated with hydropsy, a more serious disease *“pare piu gonfio che grasso” (*more swollen than fat*)*. When the pain recurred in November 1596, Paruta wrote that “*some drugs… a certain wood”* were administered more to divert hydropsy than to cure stone disease”.

The subsequent bout that kept him bedridden occured in the summer-fall of 1597. In a 1598 travel log he wrote *‘’along our journey we had bouts of podagra and chiragra*”, pointing to his great toe and hands. Crippling arthritis was associated with nephritis. In 1604 he had podagra, lost his appetite and suffered from insomnia. Fasting and increased physical activity were of little or no help. For increased activity he took trips to Frascati. His skin color changed. His pallor suggests anemia likely due to chronic kidney disease. His physician precribed a diet, hydration, and walking, which proved somewhat beneficial. In his last months of life he had various attacks of “podagra, with insomnia and lack of appetite”. On 10 February 1605, at a meeting of the Inquisition the pope had a mild stroke with aphasia, delirium, somnolence, polydipsia and amnesia. Repeated mild strokes recurred on 20 February. The final cerebrovascular event causing death supervened on 5 March. The diagnosis was “*mal di goccia*”(cerebral hemorrhage) (von Pastor, 1958–1964).

(IV) Clement X, Pontificate: 29 April 1670–22 July 1676.

Born Emilio Altieri on 30 July 1590 in Rome and elected pope at the age of 81 (Fig. 1). He is best known for Palazzo Altieri and its original stairway in Rome (architect Carlo Antonino de Rossi) and the Fountains in St. Peter Square (architects Carlo Maderno and Gian Lorenzo Bernini).

At coronation he was still healthy, would wake-up 2 h before dawn and fall asleep after having supper two hours before sunset. Since the start of his pontificate he suffered from gout. In June 1676, dropsy was recorded for the first time, a month before his death. By mid-July, “malignant fever” supervened which led to stroke, aphasia and death on July 23. Hydropsy was listed as the cause of death (Gualino, 1934).

(V) Innocent XI, Pontificate: 21 September 1676–12 August 1689.

**Fig. 2:**
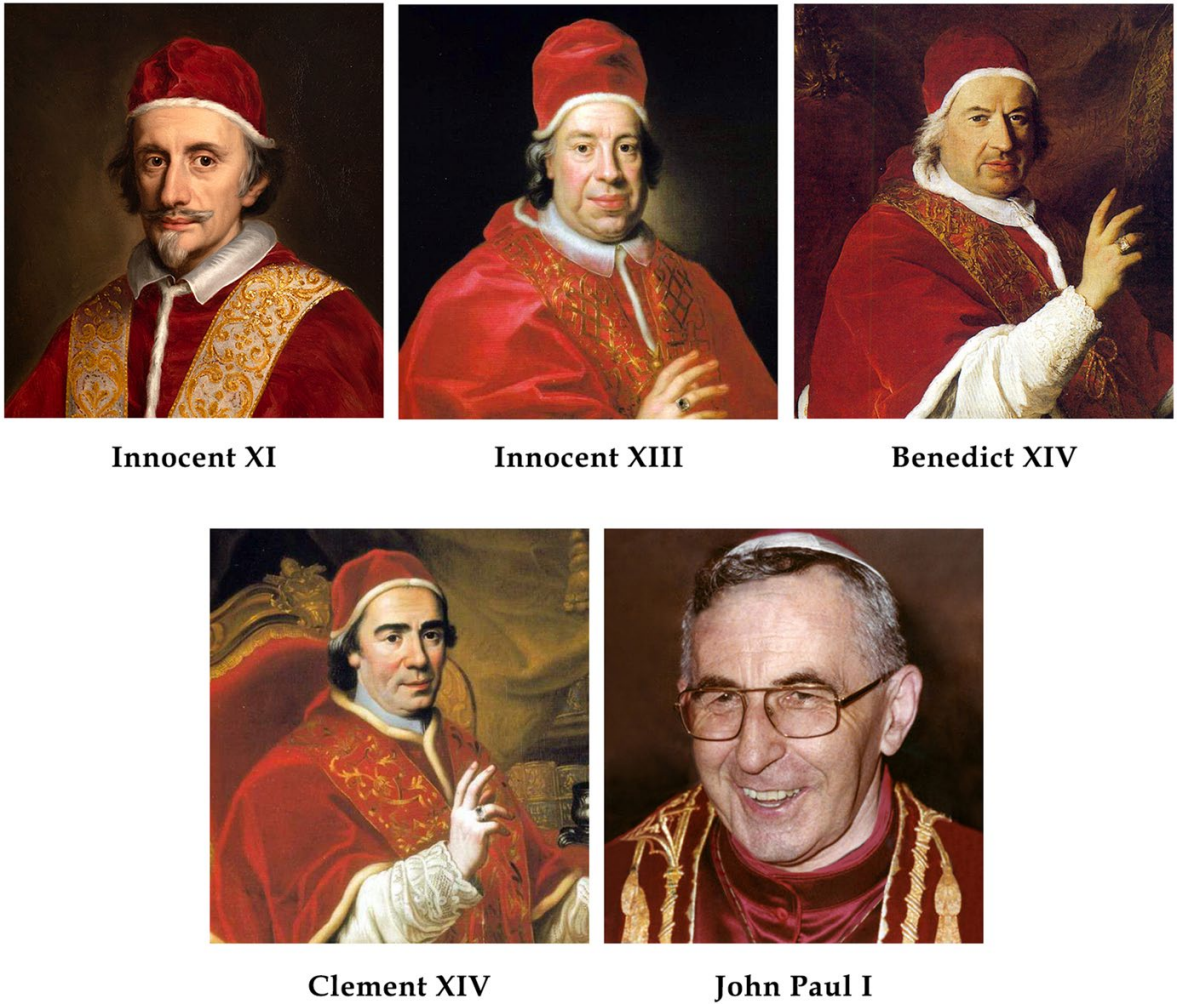
Popes reigning from 1676 to 1978

Born in Como on 5 January 1611, Benedetto Odescalchi was a tall, lean, austere, reserved, and ascetic man (Fig. 2). Devoted to prayer, work and charity, he waged a campaign against hunger. During his pontificate there were no funds available to patron the arts because of debts accumulated by his predecessors. So he lived modestly and asked the cardinals to avoid excessive expenses. He is recognized for having as his personal physician Giovanni Maria Lancisi (1654–1720), a founder of modern clinical medicine, and Professor of Epidemiology and Anatomy at the senior college of the University Sapienza in Rome.

Innocent XI suffered for 14 years from recurrent bouts of podagra. He had renal stone disease (von Pastor, 1958–1964), and agonized from undefined “symptoms of nephritis”. “His feet became edematous” and “he was obliged to remain in bed” for 2 months before death. “High fever got the best of him”. “He was bled”—without improvement—by his young archiater Giovanni Maria Lancisi who had just been nominated physician in chief at Santo Spirito Hospital. “At autopsy two small kidneys and two stones respectively of 7 and 9 oz were recovered” (von Pastor, 1958–1964).

(VI) Innocent XIII, Pontificate: 8 May 1721–7 March 1724.

Born Michele Angelo de’ Conti at Poli, Rome in 1655. He was elected pope at the age 66 (Fig. 2). Of average height and of sedentary lifestyle he made a steady descent into corpulence (Von Pastor XV, 1958–1964). Innocent XIII was affected by recurrent episodes of kidney stones. The last one started on 12 February 1724 and recurred on a daily basis. On 29 February 1724 (8 days before death), dropsy was entered into his medical report and confirmed again 2 days later. He died on 7 March (von Pastor, 1958–1964), probably from post-obstructive renal failure due to kidney stones.

(VII) Benedict XIV, Pontificate: 17 August 1740–5 March 1758.

Born in Bologna as Prospero Lambertini on 31 March 1675. As pope he added to the University of Rome the chairs of mathematics and chemistry. He left his library to the Institute of Science at the University of Bologna where he also instituted the first chair of surgery (Maxwell-Stuart, 1997). He was a man of medium height and built with a tendency to overindulge. Prospero was elected pope at 65 years of age, after a Conclave lasting 6 months and requiring 255 votes. He was healthy at that time and followed a strict diet. He usually drank tap water, and just a few sips of Montepulciano wine at the end of lunch. In the evening he would skip supper altogether, preferring to sip cinnamon flavored water. He loved physical activity and enjoyed meandering through the city. In his later years he became accustomed to walking in his apartments and traveling by coach. Despite insomnia, he was in excellent health until he turned 75 years old. In 1749 he began suffering from recurrent bouts of gout sometimes associated with fever that forced him to stay in bed and walk with a cane. In December of 1757 “nephritis” was entered in his medical records for the first time. But he improved and his physician noted “a reduction of the leg edema”. However, he soon took a turn for the worse, being affected by “disruptive wheezing due to hydrops of the chest”. “He was bled twice, and his breath improved”. However, “urine suppression supervened, and bladder catheterization was needed”. “His urine became purulent” leading physicians “to suspect kidney gangrene”. “Kidney disease slowly drained the strength of the old man”. He died feverish with pneumonia and bloody sputum (von Pastor, 1958–1964).

(VIII) Clement XIV, Pontificate:19 May 1769–22 September 1774.

Born Antonio Genganelli on 31 October 1705 in Sant’Arcangelo. He was elected pope while in good health at 64 years of age (Fig. 2). He is best remembered for dissolving the Society of Jesus and by the brief *Dominus ac Redemptor* of 21 July 1773.

He fought against his tendency to gain weight by horseback riding and taking long walks at a fast pace through the city. With advancing age, he would take daily walks in his apartments and traveled about by coach. Still and despite following a strict dietary regimen he gained weight. He continued horseback riding and taking walks as much he could. He suffered from insomnia, and bladder pain. In addition, he was afflicted by a leprosy-like skin disease which could have been impetigo, or a psychosomatic disease due to depression, that was “cured with high doses of mercury that triggered toxic deleterious effects in various organs”. There was concern of dropsy on 9 August 1757 when “the skin disease improved, but the humor moved into the interior of the body and caused hydropsy”. On 10 September he had an episode of syncope, followed by fever that required bleeding. He became increasingly hydropic and died of dropsy. The final cause of death recorded was acute pneumonia. In the final stage, urine suppression supervened that was followed by death within 3 days. Autopsy revealed accumulation of fluid in the abdominal cavity, aortic dilatation near its origin, and lung inflammation. “Very probably in the final days, dropsy and pneumonia supervened” which were considered enough to cause his death. There was no need to invoke poisoning (von Pastor, 1958–1964). His final days were horrific. He appeared malnourished and listless. On 8 September, he had a transient ischemic attack and was bled. On 19 September, 3 days before death bloody urine was noticed followed by urine suppression. He died 3 days later in a state of unresponsive wakefulness. Autopsy confirmed bilateral lobar pneumonia, with lungs full of blood (von Pastor, 1958–1964).

(IX) John Paul I, Pontificate: 26 August 1978―28 September 1978.

Born Albino Luciani on 17 October 1912 at Canaled’Agordo in the Province of Belluno. He was nominated Cardinal Patriarch of Venice by pope Paul VI in 1973. The day Paul VI died (6 August, 1978) Cardinal Albino Luciani was resting at the House of Ancelle del Signore della Carità at Lido in Venice because of “poor circulation in the lower limbs” attended in secret by his personal physician Francesco Da Ros, as we learn from the detailed monograph of M. Roncalli (2012). He had been admitted to hospital on many occasions, had four surgeries (tonsillectomy in 1923, adenoidectomy in 1924, cholecystectomy in 1964 and hemorroidectomy in 1965) and recovered from chronic tuberculosis that had affected him since seminary. He also took blood pressure medication for years. He was nominated pope in a conclave that lasted 26 h and at the fourth vote with nearly unanimous consent (Fig. 2). Cardinal Colombo of Milan said that “although so vital in public appearances Pope John Paul I was very ill”. He followed a strict schedule. He would wake up at 5 with a cup of coffee. At 12.30 he ate lunch. Then he rested briefly and walked in the roof garden on the 6th floor of the apostolic palace. Dinner was served at 7.30 P.M. and then it was off to bed by 10 P.M. At the Vatican he would walk after lunch. “An hour of air like prisoners” he was fond of saying. On 14 September he was seen “suffering and worried” and “complained of swollen legs”. “The old disturbance recurred. His legs were so swollen that it was “impossible to wear shoes” as the pope told Cardinal Villot. “His appearance changed. During mass he was noted to be pale with sweat pouring off of his forehead”.

On 23 September Dr. Da Ros came to the Vatican on an errand with the archiaters and noticed “slight edema, perhaps due in part to the use of heelless slippers”.

On Tuesday 27 September, he held a general audience with patients, nurses and physicians. He said “Let’s wish the best to the ill people who are gathered here, let us wish them good health. Let’s commend them to their families and caregivers. They will provide care. I was admitted to hospital eight times, and underwent four surgeries” (Roncalli, 2012).

A dialogue of John Paul I with VincenzaTaffarel his assistant nurse nun on 26 September 1978 is revealing.

“ Holy father your hands are swollen”.

“Not just my hands, but also my feet. They act like two dead weights that anchor me to the floor”

“Shall we call your personal physician or someone here at the Vatican?”

No, that won’t be necessary. Dr Da Ros was recently here and found that my heart is fine”.

“Holy father your heart has always been strong, but the danger is your blood pressure. Do you sleep at night?”.

“Lately I’ve been waking up between 2 and 3 in the morning and I read till it is time to rise. I have so many things to do”.

That afternoon the pope took a rest and walked around the apartment”.

“At which point the pope had angina with sense of oppression. Sister Vincenza administered a sublingual medication”. “This event was recalled 10 years later” (Roncalli, 2012).

He had dinner and at 10 pm, bid good night to his collaborators who left his room, asking him to ring should any problems arise. He was found dead the next morning, probably by Sister Taffarel who would bring the usual cup of coffee at 5 a.m. After 10 min seeing that the coffee had not been touched, Sister Vincenza called out to the Holy Father, but received no answer. Then she entered the papal bedroom and found him expired. He had probably fallen while moving from his desk. He was dressed with difficulty because of some rigor mortis had set in. Professor Buzzonetti assistant to archiater Professor Fontana “found him cold and certified sudden death at 11 pm caused by myocardial infarction”. No autopsy was requested. The pope had died alone, because of the weight of the tiara and “his unwillingness to bother his staff” (Roncalli, 2012).

## Results

Nine popes who reigned in the years 1559–1978 died with dropsies from various causes (Table 1). Their mean age at the start of their pontificate was 68.5 years, their mean age at death was 75.5 years. The shortest pontificates were those of John Paul I (33 days) and Innocent IX (62 days). The longest was that of Paul IV who reigned for over 24 years.

Innocent XI and Innocent XII were obese, sedentary and their corpulence increased over time. Benedict XIV and Clement XIV were robust with a tendency to obesity, but controlled their weight by following strict dietary regimens and skipping supper. Clement VIII was robust, a peripatetic walker, and horseback rider. Paul IV, Innocent IX, and John Paul I were selective eaters and long-distance walkers. Innocent IX ate barley soup for breakfast and worked straight through until supper, which was frugal.

Gout and chronic kidney disease most likely account for the edema in Clement VIII, Clement X, Clement XI, Benedict XIV, and Clement XIV. Kidney stone disease caused the acute obstructive nephropathy and edema of Clement XIII. Toxic nephropathy probably caused the renal disease and edema of Benedict XIV, severe malnutrition that of Innocent IX and hypertension, heart failure, and chronic kidney disease that of John Paul I. Final causes of death included syncope, stroke, pulmonary edema, pleural effusion (Table 2).

The time elapsed between the first appearance of edema and death is shown in Table 3. Edema was diagnosed 45 days before the death of Clement XIV, 8 days before the death of Clement XIII, 14 days before the death of John Paul I, 1 month before the death of Clement X, 55 days before the death of Innocent X. After the onset of edema, 5 months elapsed before the death of Benedict XIV, 6 months in that of Paul IV, and 10 years for Clement VIII. Innocent XI, Benedict XIV, Clement XIV underwent therapeutic bleeding because of edema.

**Table 2:** Etiology of edema and final causes of death of edematous Popes

Pope	Etiology of edema	Possible causes of death
Paul IV	Gout, CKD, liver cirrhosis	Old age, CKD, anemia, heart failure
Innocent IX	Malnutrition	Acute pneumonia, stroke
Clement VIII	Gout + kidney disease	CKD, anemia and stroke
Clement X	Gout + kidney disease	CKD, generalized edema
Innocent XI	Gout + kidney disease	CKD
Innocent XIII	Kidney Stone Disease, peritonitis, AKI	Urinary obstruction,
Benedict XIV	Gout,CKD, leg edema, pleural effusion, anuria	Acute pneumonia, AKI
Clement XIV	Obesity, skin lesion, mercurials, CKD	Acute pneumonia + ascites (at autopsy)
John Paul I	Hypertension, heart failure	Myocardial infarction

**Table 3:** Time elapsed between first diagnosis of edema and death of edematous popes

Pope	Time to death after diagnosis of edema
Paul IV	6 months (25 February to 18 August)
Innocent IX	55 days (6 November to 30 December)
Clement VIII	10 years
Clement X	1 month
Innocent XI	27 days
Innocent XIII	8 days
Benedict XIV	6 months
Clement XIV	45 days (9 August –22 September 1774)
John Paul I	14 days officially, hidden for many years

## Discussion

### On Edema and Diuretic Plants: a Historical Appraisal

As an external sign, edema, its diagnosis, and treatment have been an integral component of the history of humankind since time immemorial. In the Edwin Smith surgical papyrus, edema was represented by a wavy-line hieroglyphic similar to that of the water waves of the flooding Nile (Breasted, 1930). Edema was carefully described along with hydrops in *The Corpus Hyppocraticum* (Touwaide & De Santo, 1999).

Affected individuals usually consult a physician who after inspection and palpation makes the diagnosis. “Compression of the skin with a finger often results in pitting”; this can be a useful aid in assessing the severity of edema and its type. Managing edema has long attracted the interest of scientists and physicians. Until 1923, when Alfred Vogl (1896–1944) introduced organic mercurial diuretics treatment was based on drugs of limited effectiveness that were derived from plants. (Eknoyan, 1997). The Bible describes a total of 206 plants, some as food others as drugs, 95 of which have been identified, and 32 of those have diuretic properties (Table 4) (Aliotta et al., 2016).

**Table 4:** Diuretic plants of the Bible (From Aliotta et al. 2016)

*Allium cepa* L. Onion; *Allium porrum* L. Leek; *Allium sativum* L. Garlic
*Amygdalus communis* L.(Almond)
*Anethum graveolens* L. (Dill)
*Artemisia herba-alba Asso *(White Wormwood)
*Astraragalus gummifera* Labill. (Locoweed resin)
*Atriplex halimus *L. (Mallow)
*Brassica nigra *L.(Black mustard)
*Capparis spinosa *L. (Caper)
*Ceratonia siliqua *L. (Carob)
*Cicer arietinum* L. (Chickpea)
*Cinnamomum cassia* Blume (Cinnamon)
*Cinnamomum zeylanicum* Nees (Ceylon Cinnamom)
*Cistus incanus* L. (Rockrose, Labdanum)
*Citrullus colocynthis *(L.) Schrad. (Gourd, Wild Colocynth)
*Commiphora gileadensis* L. (Balm)
*Coriandrum sativum *L.(Coriander)
*Crocus sativus *L. (Saffron)
*Cucumis melo *L. (Cucumber)
*Cuminum cyiminum *L. (Cumin)
*Cuprersuss sempervirens *L. (Italian Cypress)
*Eruca sativa *Miller (Rocket)
*Ficus carica *L. (Fig)
*Hordeum vulgare *L. (Barley)
*Juglans regia *L. (Walnut)
*Juniperus communis *L*. *(Juniper)
*Malva sylvestris *L. (Mallow)
*Myrtus communis* L. (Myrtle)
*Nigella sativa* L. (Black Cumin)
*Olea europaea* L. (Olive)
*Phoenix dactylifera* L. (Date Palm)
*Populus alba* L. (White Poplar)
*Ruta chalepensis* L. (Rue)

The founder of Western botany is considered to be Theophrastus (370–285 BC), who succeeded Aristotle at the Lyceum in Athens in 323 BC. The botanical works of Theophrastus, *Historia Plantarum* and *De Causis Plantarum*, have been preserved intact. They represent the apex of ancient thought on how to deal with almost every aspect of plant therapy. Pedanius Dioscorides (ca. 41–79 AD) described edema in 47 passages of his *De Materia Medica*. He identified 105 plants with diuretic properties and listed 23 plants with cathartic effects that could be used to treat dropsy. They are exemplified by figs, cucumber, squill and wild vine (De Matteis Tortora 1994; Touwaide et al., 1997, 2002, 2005). Recent experimental studies in man and animals have confirmed that Disocorides was quite correct in his reporting of the potential diuretic properties of several plants (Yarnell &Touwaide, 2019).

Pliny the Elder (23–79 AD), Admiral of the Roman imperial fleet anchored at Misenum who died of asphyxia during the rescue operation of Pompeii and Herculaneum during the eruption of Vesuvius in 79 AD had authored *Naturalis Historia* wherein he dedicates considerable space to diuretics. He discusses no less than 2000 manuscripts and made full use of Theophrastus ‘ Botany. Pliny’s Books XXIII–XXVII deal with remedies originating from plants (De Santo et al., 1989); 86 of which had diuretic action and were used to treat dropsy (Aliotta & Pollio, 1994). It is noteworthy that six of them *Ceratonia siliqua*, *Crocus sativus*, *Cyperusesculentus*, *Pimpinella anisum*, *Urginea maritima*, are included in a list of very useful plants compiled by the World Health Organisation (Penso, 1986).

Joseph Jacob Plenck (1735–1807) is considered the forerunner of modern European dermatology and diuretic therapy (Aliotta et al., 1994). He served as professor of surgery, botany and mineralogy and secretary for life of the Military Medical Academy of Vienna (1786–1807). Plenck wrote in Latin and German *Icones Plantarum Medicinalium secondum Sistema Lynnaei cum enumeration virum et ususmedici, chirurgici et diaetetici*, Viennae (apud Rudolphum Graeffer et Soc., 1788–181). A treatise in folio illustrating the therapeutical virtues of 752 plants classified according to Linneus that was used in all military hospitals of the Holy Roman Empire. Plants were illustrated thoroughly. For each plant he gave name, class, order, types, species, place of origin, and the pharmacologic effects. A total of 111 plants (30 of them described by Dioscorides) have been identified as having diuretic properties. This contribution is currently recognized in many Pharmacopeas (Aliotta et al., 1994; De Santo et al., 2002). Two of the described plants, squill and foxglove, turned out to be effective in edematous patients with heart failure and continue to be used until the present (Aliotta et al., 2004; Withering, 1785). This was the armamentarium that was used for all popes before John Paul I. It should be emphasized that Benedict XIV a gouty person with chronic kidney disease became edematous and used to drink all the day cinnamon flavored water a diuretic plant recommended by Dioscorides (Table 4).

### Classification of Edematous States

Edema is ever present in biomedical literature; however, the paucity of current data available on elderly patients makes it unsuitable for any discussion of dropsies in 9 elderly popes (Tables 1, 2, 3) who started their pontificate at a mean age of 66.3 years and died at a mean age of 75.4 years. Thus, particular care was devoted in developing Table 5 based on recent reports specifically devoted to the elderly (Acquah et al., 2019; Bolliger & Franzech, 1992; Pomero et al., 2017; Thaler et al., 2010). The latter is an exhaustive review.

**Table 5:** Edema in the elderly. Based on data of Bolliger & Franzech (1992), Thaler et al. (2010), Pomero et al. (2017), Acquah et al. (2019)

*A. Generalized edema*	
Heart disease	
Liver diseases	
Kidney disease	
Nephrotic	
Nephritic	
Acute glomerulonephritis	
Acute tubular necrosis	
Chronic kidney disease (CKD3-4)	
Patients on dialysis (CKD5)	
Pulmonary Hypertension	
Sleep apnea	
Chronic lung disease	
Hypothyroidism	
Malnutrition	
Obesity	
Pharmacological agents	
Antihypertensive agents	
Glitazones	
Hormones (Corticosteroids, Estrogen, Progesterone, Testosterone)	
Other ( Glitazones, Non Steroidal Anti Inflammatory Agents)	
Typical of the intensive care unit	
Multiple Organ Dysfunction Syndrome	
Burns	
Cerebral edema	
Infections	
Stroke	
Trauma	
Tumors	
Typical in women	
Idiopathic	
Premestrual	
Pregnancy	
Preeclampsia	
*B. Localized edema*	
Gravity associated edema	
Long standing, sitting in a plane or in a car, being bedridden	
Return to microgravity from space flights	
Venous Insufficiency	
Deep vein thrombosis	
Chronic vein insufficiency	
Reflex sympathetic dystrophy	
Lymphedema	
Malignancies	
Soft tissue infection	
Animals bites	
Cellulitis	
Erysipelas	
Filariasis	
Impetigo	
Necrotizing fascitis	
Trauma	
Lipedema	

Richard Bright (1789–1858) reported on the association of edema and proteinuria in acute and chronic nephritis (Bright, 1827) . It was Domenico Cotugno (1736–1822) who the first described the association of edema and proteinuria in his 1770 *De ischiade nervosa commentaries* (De Santo, Cirillo, et al., 2001; De Santo, Pollastro, et al., 2001). but did not link it to kidney disease*.* Fluid overload is present in nearly 50% of patients in the late stages of CKD that correlate with increasingly reduced glomerular filtration rate (GFR). It is associated with hypertension, congestive heart failure, left ventricular hypertrophy, and edema. The treatment of hypertension has emerged as an important therapy of those patients. (Hung et al., 2014; Vasavada & Agarwal., 2003; Adnan et al., 2016).

It is not known when measurements of blood pressure began in the Vatican palaces. Probably it happened at the beginning of the twentieth century, after Scipione Riva Rocci (1863–1937) developed at Pavia a sphygmomanometer for the easy measurement of blood pressure. Its use in association with a phonendoscope gave the possibility of utilizing the auscultatory method proposed by Nikolai S. Korotkoff in 1906. Much can be gleaned on hypertension management in the twentieth century from the narrative of John Paul I. He was the typical hypertensive patients who ended in heart failure and chronic kidney disease and probably became edematous because of his excessive salt intake.

### On Papal Edema

Data in Tables 1, 2 and 3 demonstrate that edema of various causes affected nine popes reigning in the years 1555 to 1978. It was a bad prognostic sign foreboding imminent death in 8 out of 9 cases. Etiology included heart failure, cirrhosis of the liver, malnutrition,CKD, and obstructive nephropathy.

Edema was caused by hypertension, heart failure, and chronic kidney disease in John Paul I. Cirrhosis of the liver disease chronic kidney disease and anemia affected Paul IV. Chronic kidney disease from gout caused edema in Clement VIII, Clement X, Innocent XI, Benedict XIV. Chronic kidney disease due to mercurial toxicity caused edema in Clement XIV, postrenal acute kidney injury due to stone obstruction and sepsis caused edema of Innocent XIII. Paul IV also had deep vein thrombosis as attested by one edematous leg for which he was prescribed purges to drain the fluid. Three popes (Innocent XI, Benedict XIV, Clement XIV) were bled to reduce impending pulmonary edema.

Edema in CKD is associated with excessive intake of sodium chloride. (Kurtzman, 2001). However, we know that in all stages of CKD, there is increased body fluid volume (Tsai et al., 2014). It is likely that the edema of all the popes was facilitated by the sodium-rich diet of the upper classes in those days. Of note is the principal cause of kidney disease as obstructive nephropathy due to gout or stones. It has been shown that many popes suffered of kidney stone disease of gouty and non gouty origin, the former was more frequent (De Santo et al., 2021a, b), however the last gouty pope was Pius X who died in 1914. Thus in the last hundred year no pope was gouty indicating that gout is no longer a diseases of wealthy people. This paper does not aim to provide recommendations for the future care of Catholic pontiffs; however, any cardinal entering the conclave should be mindful of debilitating disease as well as of the stress imposed by the papal tiara. Pope John Paul I is a good example as he had a long-lasting disease incompatible with the vitality requested by his pontifical role and validates the underlying premise of the classic epidemiologic transition theory (Omran, 1971). It has been shown that people with wealth and education are not unlike poor uneducated people in adopting excessive lifestyles causing noncommunicable chronic diseases associated with morbidity and mortality (Caldwell, 2001; Marmot et al., 1991; McKeown, 2009; Omran, 1971; Pearson, 2003). However, wealthy educated people when made aware of the risk connected with those diseases adapt their lifestyles, whereas poor uneducated people may not be able to afford that luxury. Thus, the latter group will experience morbidity and mortality of the disease where rich, well-educated people can achieve prevention. The theory also explains why gout disappeared from the Vatican palaces but increased in the general population (De Santo et al., 2021a, b, 2022). We are certain that the use of salt in the papal kitchen is no longer what it once was in the past. Sadly, the case of John Paul I indicates that the pope died in loneliness.

The paper has several strengths and a limitation. Strength is provided by the recorded details of edema (where, when, length of time) at the time of death, and its clinical picture of the popes.

### Study Limitations

Its limitation is that the cause of edema are evident in some narratives (for example that on John Paul I) but is less evident and remains speculative for other popes.
