# Correction: The Dropsy of Popes (1555–1978): A Bad Prognostic Sign Foreboding of Death

**DOI:** 10.1007/s10943-022-01599-1

**Published:** 2022-06-11

**Authors:** Natale Gaspare De Santo, Carmela Bisaccia, Luca Salvatore De Santo

**Affiliations:** 1 Università Della Campania Luigi Vanvitelli, Naples, Italy; 2 Mazzini Institute Naples, Naples, Italy; 3 Division of Heart Surgery, University of Campania Luigi Vanvitelli, Naples, Italy

## Correction to: Journal of Religion and Health https://doi.org/10.1007/s10943-022-01578-6

In the original publication of the article, the author noticed an error in Section, "Classification of Edematous States" under second paragrapgh, the sentence should read, "It was Domenico Cotugno (1736–1822) who the first described the association of edema and proteinuria in his 1770 De ischiade nervosa commentaries (De Santo, Cirillo, et al., 2001; De Santo, Pollastro, et al., 2001)" instead of "It was Domenico Cotugno (1736–1822) who the first described the association of edema and proteinuria in his 1770 De ischiade nervosa commentaries (De Santo, Cirillo, et al., 2001; De Santo, Pollastro, et al., 2001). but did not link it to kidney disease".

